# *Ab Initio* and Theoretical Study on Electron Transport through Polyene Junctions in between Carbon Nanotube Leads of Various Cuts

**DOI:** 10.1038/s41598-020-63363-3

**Published:** 2020-05-15

**Authors:** Yiing-Rei Chen, Ming-Kuan Lin, Dun-Hao Chan, Kuan-Bo Lin, Chao-Cheng Kaun

**Affiliations:** 1 Department of Physics, National Taiwan Normal University, Taipei, 11677 Taiwan; 2 Department of Materials Science and Engineering, National Cheng Kung University, Tainan, 70101 Taiwan; 3 Research Center for Applied Sciences, Academia Sinica, Taipei, 11529 Taiwan; 4 Department of Physics, National Tsing-Hua University, Hsinchu, 30013 Taiwan

**Keywords:** Nanoscale devices, Nanoscale materials, Theory and computation

## Abstract

In this study we look into the interference effect in multi-thread molecular junctions in between carbon-nanotube (CNT) electrodes of assorted edges. From the tube end into the tube bulk of selected CNTs, we investigate surface Green’s function and layer-by-layer local density of states (LDOS), and find that both the cross-cut and the angled-cut armchair CNTs exhibit 3-layer-cycled LDOS oscillations. Moreover, the angled-cut armchair CNTs, which possess a zigzag rim at the cut, exhibit not only the oscillations, but also edge state component that decays into the tube bulk. In the case of cross-cut zigzag CNTs, the LDOS shows no sign of oscillations, but prominent singularity feature due to edge states. With these cut CNTs as leads, we study the single-polyene and two-polyene molecular junctions via both *ab initio* and tight-binding model approaches. While the interference effect between transport channels is manifested through our results, we also differentiate the contributions towards transmission from the bulk states and the edge states, by understanding the difference in the Green’s functions obtained from direct integration method and iterative method, separately.

## Introduction

Since the discovery of carbon nanotubes (CNTs) in 1991^1^, the properties of these fascinating quasi-one-dimensional, nano-scaled materials have been extensively pursued^2–4^. Such a trend has been enhanced further as graphene was rediscovered in 2004, which brought even more attention toward all different materials based on the honeycomb carbon structure. Later as the theoretical research turned to look at the boundaries of these materials, such as edges of graphene, graphite or nano ribbons^5–11^, or finite-sized CNTs of different tips^12–15^, experiments kept up as well. Scanning tunneling microscopy studies^16–20^ on LDOS of different graphite edges have been performed, and the edge states of graphite are confirmed. However, similar experimental efforts on CNTs boundaries^21,22^ are even more challenging and yet to be solidly realized.

Due to the relatively well-defined lead-molecule covalent bonding and the finite transport channel involved, prospected nano-devices^23^ using CNTs is an alternative and interesting choice, compared with the largely studied single-molecule junctions with metal-molecule linkages^24–26^. The CNTs, proposed to serve as the junction connecting the electrodes, or the electrodes themselves, or forming a heterostructured junction, give rise to peculiar properties in transport through these devices^27–30^.

In recent years, molecular junctions employing CNT electrodes have been fabricated and measured by much more controlled and developed strategies. In some cases a single protein can be site-specifically attached to the leads^31^, while in others the linkage morphology, or the question of whether the linkage is formed by multiple molecules^32^, becomes important and therefore requires further confirmation. As the idea of a single-molecule transistor is gradually realized in these systems, the properties of the CNT leads and the details of the molecule-CNT contact are both crucial for further pursuit. Interference effect^33–35^, among all the above, should accompany the formation of multi-molecule junctions in between CNT leads, and is an example that illustrates the interplay between the two roles.

In this study, we aim at understanding the assorted cross-cut or angled-cut semi-infinite CNTs and the transport through molecular junctions bridging such leads. On the one hand we use a one-parameter  tight-binding (TB) model that describes the $(pp\pi )$ hopping in the CNTs’ honeycomb network, on the other hand we perform *ab initio* calculations. With these tools we examine the surface Green’s function, whose projection onto contact sites helps to explain the transmission behavior of molecular junctions. We also look into the LDOS, the by-product of the surface Green’s function. For different cuts of CNTs we carry out the LDOS comparison that shows the dependence of edge state contribution on the type of cut. The transmission in double-threaded molecular junctions shows clearly the interference effect, namely, how the pick of contact sites, where the molecules attach to the CNT leads, affact the transport. This is an effect that has been studied for the cross-cut armchair CNTs, and now for the angled-cut armchair CNTs and cross-cut zigzag CNTs. Again, we realize that the interference is a consequence of superposition from the properly chosen even and odd channels.

## Method

### The ab initio approach

We use the geometry relaxation process implemented in the SIESTA package^36^ to optimize the CNT bulk structure, with a force criterion of 0.02 eV/*Å*, an energy cutoff of 200 Ryd, the Double-$\zeta$ plus polarization (DZP) basis, and the Ceperley-Alder local density approximation (LDA) for the exchange and correlation functional. A rectangular supercell of 20 *Å* × 20 *Å* × *a* is used, where $a$ is the lengthwise $z$-dimension of the unit cell, to be optimized. With this choice of supercell, the distance between nearest tube walls is 9 *Å*, which allows enough vacuum to pass the bond length convergence test.

In a similar way, the structures of the junctions are then relaxed. For each junction we use a rectangular supercell of 20 *Å* × 20 *Å* × *D* *Å*, where $D$ is the span of the junction. It contains 3 to 3.5 CNT unit cells from each lead, the hydrogen atoms that saturate the dangling bonds at the rims, and the bridging polyene molecule(s). Except for the carbon atoms in the CNT unit cells that sit farthest from the polyene molecules, which are kept fixed such that they hold the bulk CNT symmetry and bond lengths, all other atom positions are relaxed with no symmetry restriction.

The relaxed junction structures are then put into the *ab initio* transport calculations, which are performed by the Nanodcal package^37^ with the density functional LDA_PZ81 and the DZP basis. Non-equilibrium Green’s function (NEGF) method^38^ is adopted in this *ab initio* process, where the leads’ self-consistent field (SCF) calculation uses the bulk CNT structure, and is followed by the SCF and transmission calculation for the open system of CNT-junction-CNT. Having done the transmission convergence test, we choose a 3-primitive-unit-cell buffer layer to sit at both sides of the scattering region, to form the linkages between the junction and the leads. Note that the Brillouin zone is sampled by a 1 × 1 × 200 Monkhorst-Pack grid, while a supercell of 30 *Å* × 30 *Å* × *D* *Å* and a corresponding real-space grid of 450 × 450 × 15 *D* are used to ensure enough vacuum and accurate transmission calculations performed by Nanodcal.

### The tight-binding (TB) approach

Theoretical results shown in this article are derived from a single-parameter model, where a uniform ($pp\pi$) hopping energy $t$ is considered for all the C-C nearest neighbor links in the entire CNT leads, the polyene molecule(s), and the coupling between the molecules and the leads. We also consider the on-site energy on all carbon atoms to be a constant that equals the CNTs’ Fermi energy ${E}_{{\rm{F}}}$, and ${E}_{{\rm{F}}}\equiv 0$.

**Figure 1:**
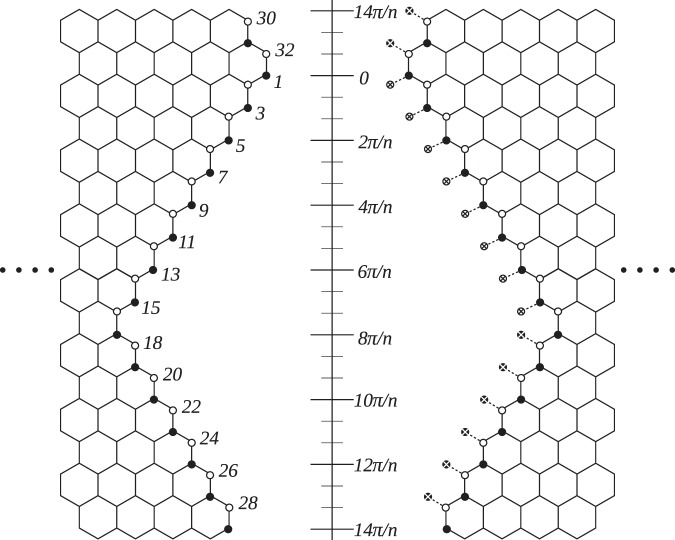
Labeling of the sites on the rim of an angled-cut $(n,n)$ CNT electrode, the correspondence to angle $\theta$, and the illustration for nodes (circles with a cross inside). Here we literally use $n=8$ to illustrate the labeling. The black dots and the white dots represent sites from two different graphene sublattices. Angle $\theta$ is also used to describe the relative positions of two contact sites, when two polyenes are in parallel and bridging the electrodes.

**Figure 2:**
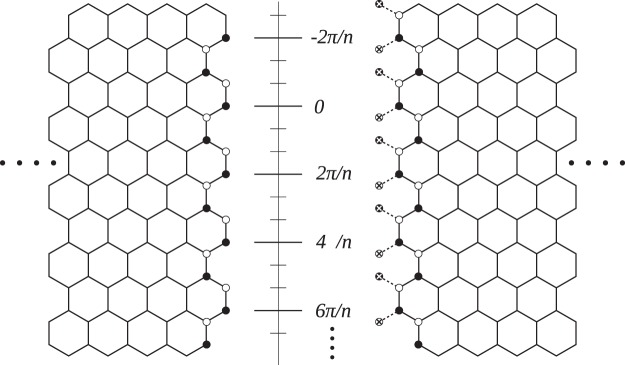
On the rim of a cross-cut $(n,n)$ CNT electrode, the correspondence of relative positions to angle $\theta$, and the illustration for nodes (circles with a cross inside).

**Figure 3:**
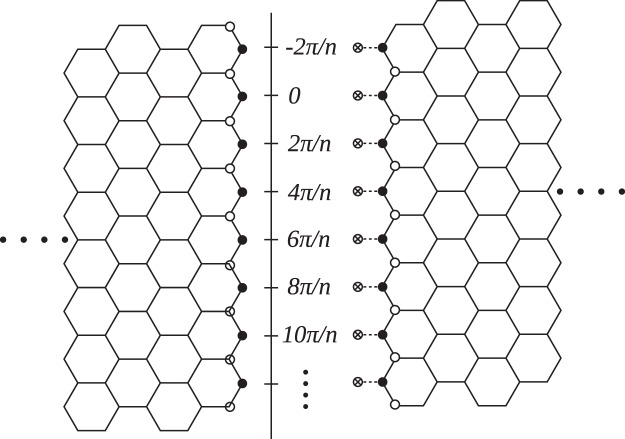
On the rim of a cross-cut $(n,0)$ CNT electrode, the correspondence of relative positions to angle $\theta$, and the illustration for nodes.

We calculate the surface Green’s function of the electrodes with the TB Hamiltonian described above, via two different paths:

(i) **The iterative method**: All CNT leads considered here have the semi-infinite symmetry. Based on such a symmetry, and this symmetry alone, one gets the self-consistent formula:
  $${g}_{s}(E)={[\alpha -{\beta }^{\dagger }{g}_{s}(E)\beta ]}^{-1}$$
  where $\alpha =E-h+i\eta$ and $h$ is the Hamiltonian of the surface layer (i.e., the unit cell at the rim), $\beta$ describes the coupling between the surface layer and the bulk (what is left as the surface layer is peeled off), and ${g}_{s}$ is the surface Green’s function.
(ii) **The integration method**: Out of the electronic states of the infinite 2-D graphene, one finds the allowed $k$-point lines in the Brillouin zone, for any specific $(n,m)$ indices that describe the chirality of the CNT of interest^3^. By doing so one obtains the $(n,m)$ CNT band structure. Any specific cut of the $(n,m)$ CNT determines a specific set of boundary conditions, namely, the nodal rim that defines the cut. From the pool of the $(n,m)$ CNT’s electronic states, we construct all possible linear combinations that give states vanishing at the nodal rim. These linearly combined states, satisfying the boundary condition of the cut, lead to the expression for the surface Green’s function of the cut. For the angled-cut armchair CNTs, e.g., the real space (**r**, **r**′) projection of the surface Green’s function is

$$\begin{array}{rcl}{g}_{s}(E,k;{l}_{1}{s}_{1},{l}_{2}{s}_{2}) & = & \mathop{\sum }\limits_{{\lambda {\prime} }_{k}{\lambda {\prime} {\prime} }_{k}}^{4n}\,\langle {l}_{1}{s}_{1}|{\lambda {\prime} }_{k}\rangle \langle {\lambda {\prime} }_{k}|\\  &  & \times \,P\,\mathop{\sum }\limits_{{\lambda }_{k}}^{4n}\,\frac{|{\lambda }_{k}\rangle \langle {\lambda }_{k}|}{E-{\lambda }_{k}+i\eta }P|{\lambda {\prime} {\prime} }_{k}\rangle \langle {\lambda {\prime} {\prime} }_{k}|{l}_{2}{s}_{2}\rangle \end{array}$$

where

$$P=\mathop{\sum }\limits_{{\tau }_{k}}^{2n}\,|{\tau }_{k}\rangle \langle {\tau }_{k}|,$$

and we use notations *λ*, *λ*′ and *λ*″ to label the eigen-states of the bulk tube. The real space position is labeled by layer $(l)$ and site $(s)$. Note that the $2n$ ${\tau }_{k}$ states, are all the linear combinations of the original $4n$ states of wavevectors *k* and −*k*, composing the complete orthonormal basis that satisfies the angled-cut boundary conditions. Obviously the boundary conditions of the angled-cut armchair CNTs’ rim are more complicated than those of the cross-cut tubes, in the sense that there are $2n$ non-equivalent sites appearing as nodes on the angled-cut rim, while all nodal sites of the cross-cut rim correspond to the same boundary condition. See illustrations in Figs. 1, 2 and 3.

With this integration method, the Green’s function thereby derived comes from the bulk’s Bloch states only. In other words, evanescent waves are excluded in the integration method, while the iteration method includes everything. Our inspection via the integration method is therefore valuable in the sense that it helps to separate the contributions from the edge states and the bulk states.

## Results and Discussions

### The LDOS at the rims of differently cut CNTs

**Figure 4:**
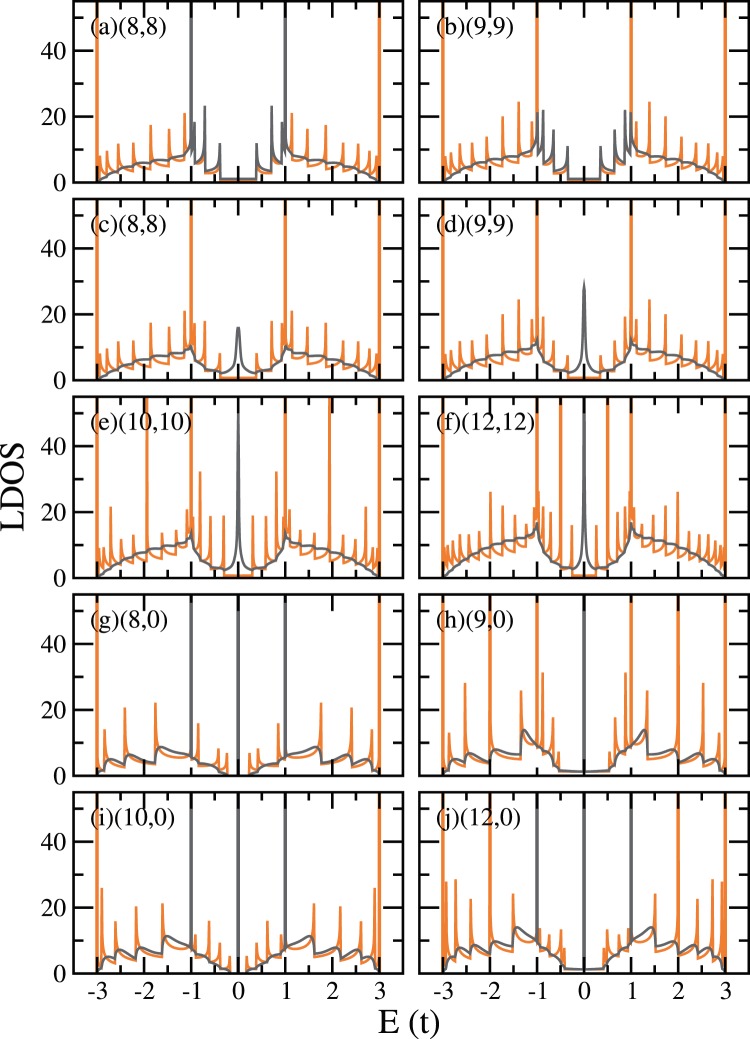
The LDOS of the $4n$ carbon atoms in the surface unit cell (grey, from iterative method), for (**a**) cross-cut (8, 8), (**b**) cross-cut (9, 9), (**c**) angled-cut (8, 8), (**d**) angled-cut (9, 9), (**e**) angled-cut (10, 10), (**f**) angled-cut (12, 12), (**g**) cross-cut (8, 0), (**h**) cross-cut (9, 0), (**i**) cross-cut (10, 0), and (**j**) cross-cut (12, 0), compared with the corresponding bulk unit cell DOS (orange). Edge states appear in all angled-cut $(n,n)$ CNTs, and all cross-cut $(n,0)$ CNTs.

The surface LDOS of selected CNTs are shown in Fig. 4. The data shown in grey are obtained from the iterative method. In order to compare with the bulk unit cell DOS (in orange), we sum up the contributions from the $4n$ carbon atoms (same number of atoms as in a bulk unit cell) of the outermost layer along the cut. As the tubes are cut, bulk features are suppressed in various ways, subject to different boundary conditions: In the cross-cut $(n,n)$ (armchair) cases only the van Hove singularities originated from $k=0$ band extrema are suppressed, while in the angled-cut $(n,n)$ cases, the boundary condition leads to entanglement among different bands, and therefore all van Hove singularities are suppressed. In the cases of even-$n$ cross-cut $(n,0)$ (zigzag) CNTs, we also see the only un-suppressed singularities at $E=\pm \,t$. This comes from the dispersionless bands in the TB model, where no other $k=0$ band extrema are present.

Moreover, for cases of cross-cut $(n,0)$ CNTs and angled-cut $(n,n)$ CNTs, considerable amount of states pop up at *E*_F_. The peak of these *E*_F_ states is wider in all cases of angled-cut $(n,n)$ CNTs, and not as singular as that in any cross-cut $(n,0)$ CNT case. The cross-cut $(n,n)$ CNTs don’t have such a peak.

**Figure 5:**
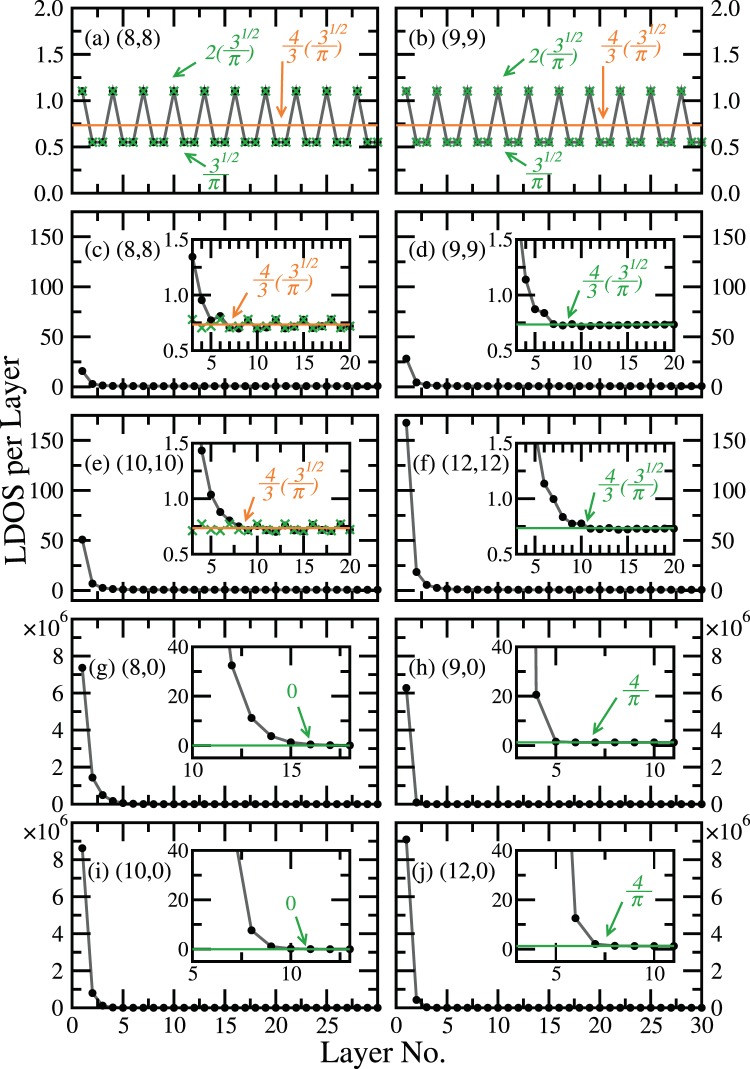
The layer-by-layer LDOS from iterative method (grey line and circle dots) at $E={E}_{{\rm{F}}}$ for (**a**) cross-cut (8, 8), (**b**) cross-cut (9, 9), (**c**) angled-cut (8, 8), (**d**) angled-cut (9, 9), (**e**) angled-cut (10, 10), (**f**) angled-cut (12, 12), (**g**) cross-cut (8, 0), (**h**) cross-cut (9, 0), (**i**) cross-cut (10, 0), and (**j**) cross-cut (12, 0). In each case the layer is sliced according to the shape of the cut, and each layer contains 4*n* carbon atoms, same as a bulk unit cell. Green lines and cross symbols present results from the integration method. When in agreement with the bulk result (orange), only the integration method result is shown.

Next we investigate the layer-by-layer LDOS, for energy at ${E}_{{\rm{F}}}$ only. Results from path (i) and path (ii) are both shown in Fig. 5, to compare with the bulk value. It is clearly seen in (c)~(j) that the peak at ${E}_{{\rm{F}}}$ is due to edge states, and this is true for all angled-cut $(n,n)$ CNTs and all cross-cut $(n,0)$ CNTs. While the LDOS of every cross-cut $(n\mathrm{,0)}$  CNT decays monotonically to the bulk value, in cases of the angled-cut $(n,n)$ CNTs with $n\ne 3m$, $m\in {\mathbb{N}}$, oscillations of a three-layered cycle emerge from the decay of the edge state. As for the angled-cut $(n,n)$ CNTs with $n=3m$, the edge state LDOS decays and converges to a single value, instead. However, the oscillating LDOS is the signature feature of all cross-cut $(n,n)$ CNTs, and all angled-cut $(n,n)$ CNTs. Even for angled-cut cases of $n=3m$, the oscillations are still vaguely seen, before the iterative LDOS fully converges to the single bulk value.

### Transmission

**Figure 6:**
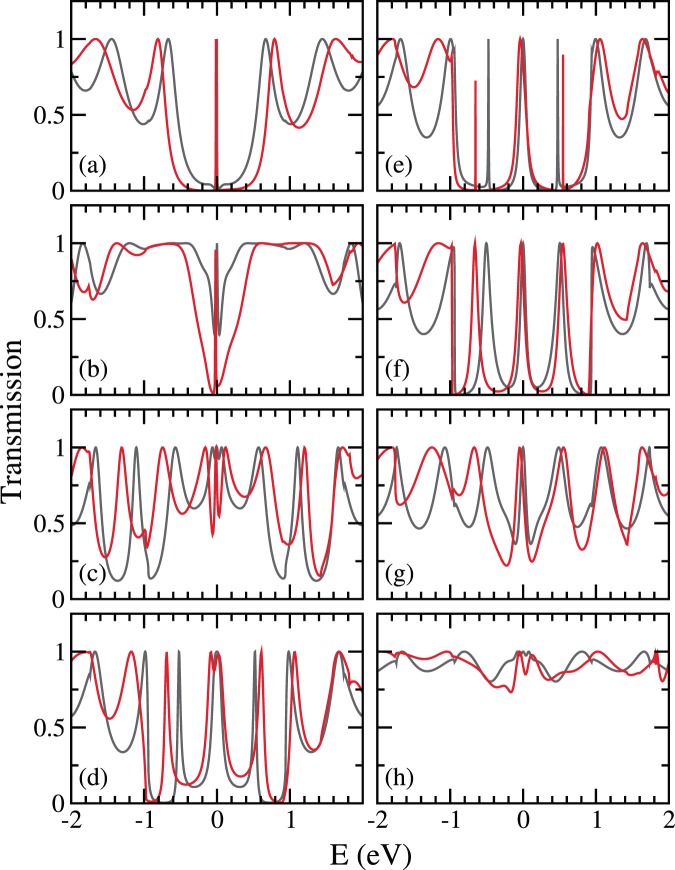
TB (iterative, in grey) and *ab initio* (red) transmission through angled-cut (8, 8) CNT leads bridged by one single polyene molecule at site (**a**) 1, (**b**) 3, (**c**) 5, (**d**) 7, (**e**) 9, (**f**) 11, (**g**) 13, and (**h**) 15. From the most out-poking to the most in-tucking, in order.

**Figure 7:**
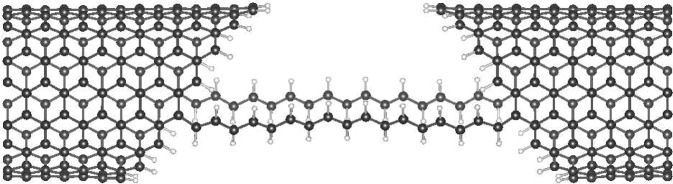
An illustration of a two-polyene junction, using contact site combination (9, 24), on the angled-cut (8, 8) electrodes.

**Figure 8:**
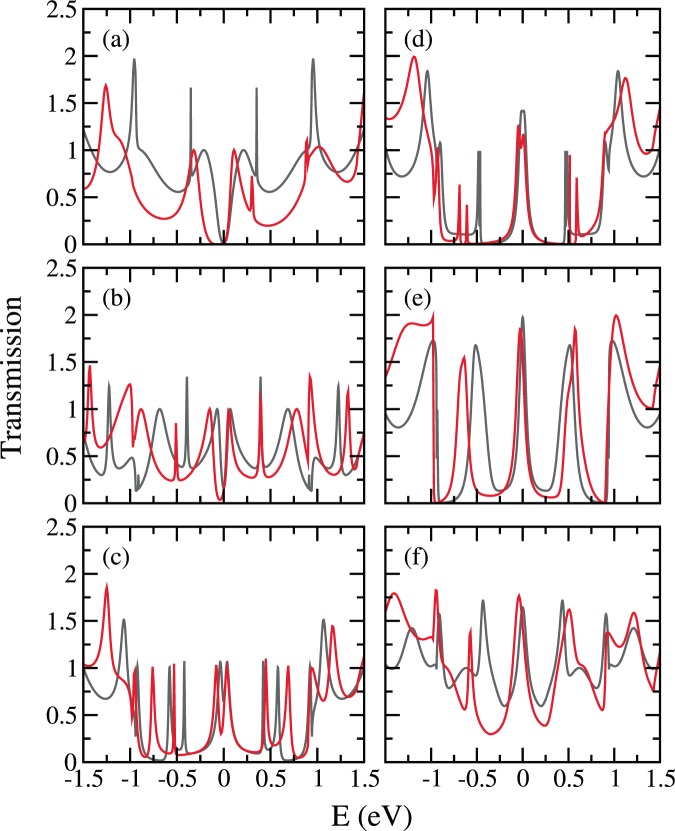
TB (iterative, in grey) and *ab initio* (red) transmission through angled-cut (8, 8) CNT leads bridged by two polyene molecules at site combination (**a**) $\theta =8\pi /3n$(3–30), (**b**) $14\pi \mathrm{/3}n$(5–28), (**c**) $20\pi \mathrm{/3}n$(7–26), (**d**) $26\pi \mathrm{/3}n$(9–24), (**e**) $32\pi /3n$(11–22), and (**f**) $38\pi \mathrm{/3}n$(13–20).

With the $n=8$ angled-cut armchair CNT leads, we consider C_17_H_19_, the 17-carbon polyene molecule(s), and perform the *ab initio* calculations for transmission through all non-equivalent one-polyene junctions (shown in Fig. 6) and selected two-polyene junctions (shown in Figs. 7 and 8), excluding two cases where the two polyenes sit too close in reality. Depending on contact sites, the gap span is tuned to allowed the C_17_H_19_ polyene to fit in, whereas the choice of C_17_H_19_ is meant to prevent the two leads to touch at their most out-poking sites, even when the polyene is bridging their most in-tucking sites. For each junction the *ab initio* result is shown on top of that from the iterative method, and the contact site combinations are labeled with the index illustrated in Fig. 1. In all cases, the transmission results from both approaches reach agreement. Especially, via either approach, the two-polyene cases clearly show the transmission’s dependence on contact combination, and therefore reveals the interference effect.

The *ab initio* central (${E}_{{\rm{F}}}$) features exhibit shifts with respect to the iterative method results. This is due to charge transfer, as discussed before in the literature^34^, and maps to the TB model as a slightly negative effective molecular on-site energy. Other than this, the most obvious discrepancy mainly lies in the energy scale, as shown by the shifts between relative off-center features from both approaches. This is discussed in the next subsection.

**Figure 9:**
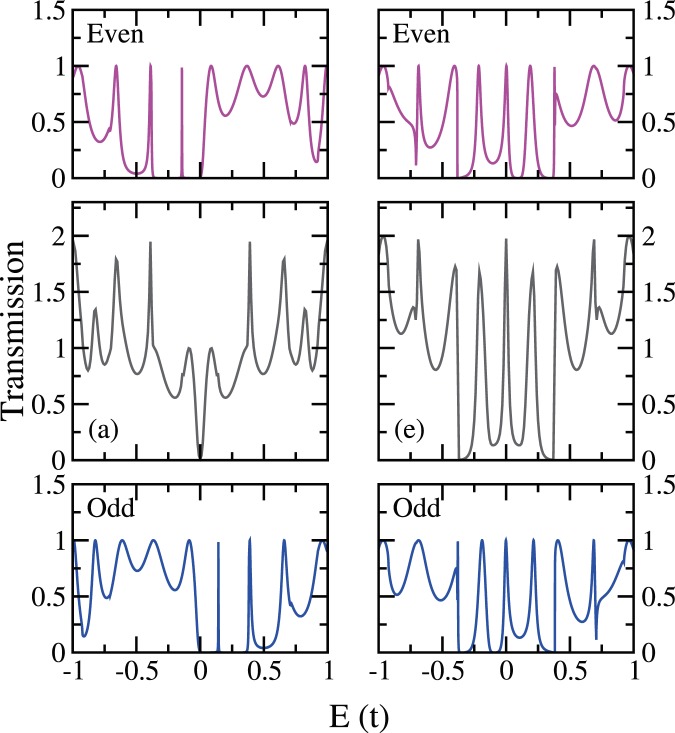
Even(crimson) channel and odd(blue) channel contribution for the transmission through angled-cut (8, 8) CNT leads bridged by two 17-carbon polyene molecules at site combination (**a**)..(3–30) and (**e**) $\theta \mathrm{=32}\pi \mathrm{/3}n$(11–22), cases selected from Fig. 8.

The 2-polyene transmission is composed of contributions from the even and the odd channels, where the even (odd) channel is the even (odd) combination of the real-space two parallel polyenes. These two channels diagonalize the effective two-dimensional subspace characterizing the junction of two threads. In Fig. 9 we use the most destructive case (a) and the most constructive case (e) from Fig. 8, to illustrate how the individual contributions from the even and the odd channels are simply summed up to give the total transmission.

### Molecular orbitals and features

Note that the hopping energy $t$ is fitted by the bulk’s band feature in the vicinity of ${E}_{{\rm{F}}}$. With this energy scale, we show the *ab initio* transmission data on top of the iterative method data. For all cases it appears that the TB polyene molecular level spacings are smaller than those in the *ab initio* version.

To see this, we do the *ab initio* calculation for polyene C_17_H_19_ in vacuum, via the SIESTA package with the same standard we use for the junctions. The HOMO and LUMO of C_17_H_19_ suggest an intra-molecular hopping that is 1.3 times the CNT hopping. As the polyenes act as the bridge in the junction, the average molecular C-C bond length increases by only 0.5%, while the standard deviation shrinks to under 22% of the vacuum value. Although the increased average and the shrunken standard deviation of bond length should bring smaller molecular level spacings, all C-C bonds of the polyene in the junction are shorter than the bulk CNT’s two different bond lengths (1.412 *Å* and 1.415 *Å*). This means, the intra-molecular hopping is still larger than the CNT hopping, even in the junction form, which explains the disagreement between the TB and the *ab initio* results on energy spacings of the molecular level resonance.

### Edge states from the difference between path (i) and path (ii)

**Figure 10:**
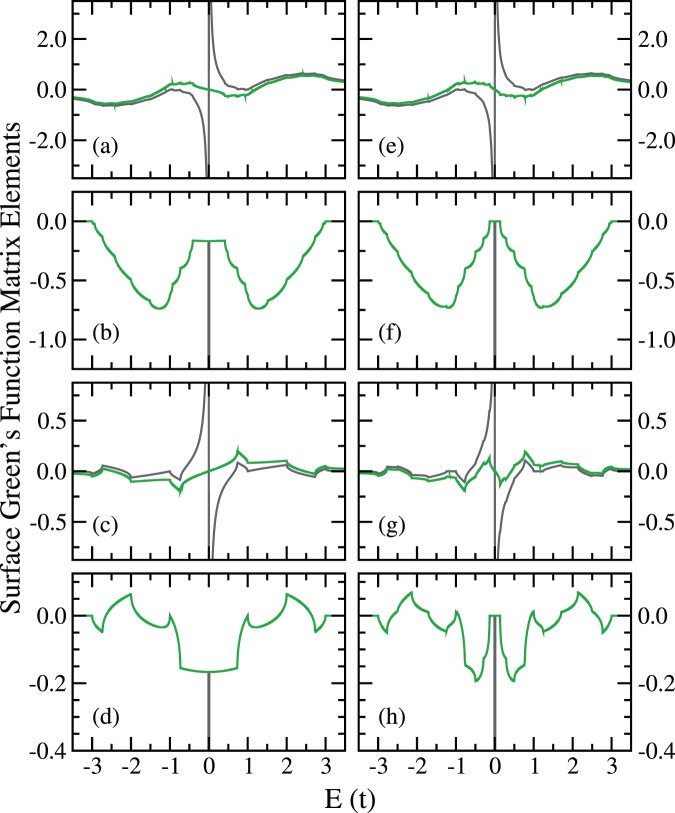
Some surface Green’s function elements, from iterative method (grey) and integration method (green), are shown for the cross-cut zigzag  CNTs: (**a**) (12, 0) Re$({g}_{s,11})$, (**b**) (12, 0) Im$({g}_{s,11})$, (**c**) (12, 0) Re$({g}_{s,17})$, (**d**) (12, 0) Im$({g}_{s,17})$, (**e**) (13, 0) Re$({g}_{s,11})$, (**f**) (13, 0) Im$({g}_{s\mathrm{,11}})$, (**g**) (13, 0) Re$({g}_{s\mathrm{,17}})$, and (**h**) (13, 0) Im$({g}_{s,17})$.

We use the two cross-cut zigzag CNTs, $(12,0)$ and $(13,0)$, to illustrate the difference between the iterative method and the integration method. In Fig. 10, results from both methods are shown for selected matrix elements of the surface Green’s function, where the site indices follow the labeling illustrated in Fig. 3. The disagreement occurs at the vicinity of ${E}_{{\rm{F}}}$ for all cases shown.

**Figure 11:**
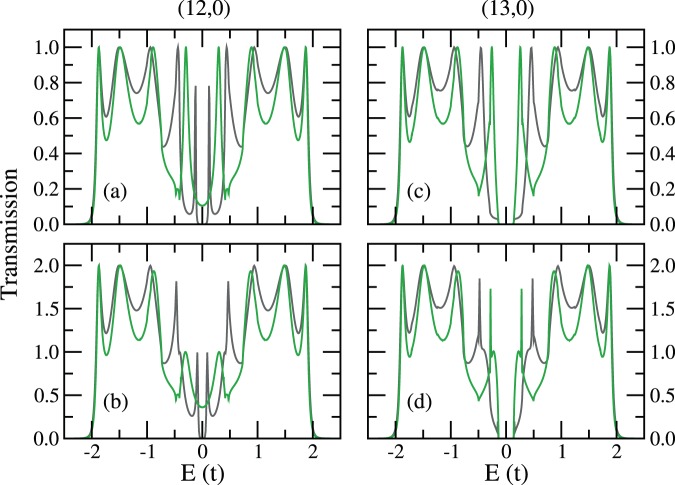
TB transmission through cross-cut zigzag CNT leads bridged by parallel 8-carbon polyene(s), from iterative method (grey) and integration method (green): (**a**) (12, 0) CNT leads and one-polyene junction, (**b**) (12, 0) CNT leads and two-polyene junction of site combination (1, 7), (**c**) (13, 0) CNT leads and one-polyene junction, (**d**) (13, 0) CNT leads and two-polyene junction of site combination (1, 7).

With the detailed energy dependence of the surface Green’s function matrix elements readily calculated, we then consider C_8_H_10_, the 8-carbon polyene(s) as the junction, and investigate the one-polyene case and the two-polyene cases, as shown in Fig. 11. Note that the reason of choosing a polyene species with even number of carbon atoms is inevitably the geometry of the cut, and such a choice gives no molecular level at ${E}_{{\rm{F}}}$. For the cross-cut $(13,0)$ leads, the TB transmission results from both methods echo the gap of the bulk CNT, in other words, the edge states at ${E}_{{\rm{F}}}$ does not make up for the gap, but does modify the transmission, especially near both band edges. For the cross-cut $(12,0)$ leads, the original un-cut bulk $(12,0)$ CNT is a semi-metal. However, the transmission in the vicinity of ${E}_{{\rm{F}}}$ given by the integration method is taken away by the edge states.

**Figure 12:**
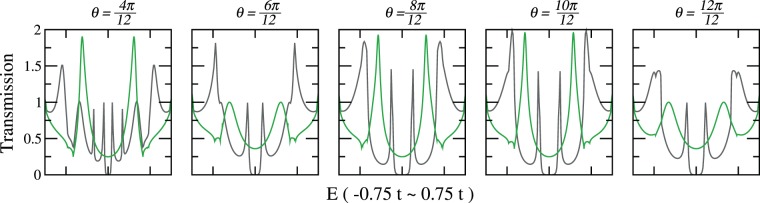
TB transmission through cross-cut (12, 0) zigzag CNT leads bridged by two parallel 8-carbon polyenes, from iterative method (grey) and integration method (green), with contact site combinations $\theta =4\pi /12$(1, 5), $\theta =6\pi /12$(1, 7), $\theta =8\pi /12$(1, 9), $\theta =10\pi /12$(1, 11), and $\theta =12\pi /12$(1, 13).

In Fig. 12 we show the transmissions of all 2 polyene cases (except contact combination (1, 3) that is geometrically too close for the two parallel polyene threads) with the cross-cut (12, 0) leads. The results from the iterative method and the integration method give the transmissions with and without the edge states. Within each individual method, the results from different contact combinations show the effect of interference.

## Conclusion

We present in this article a further study for the interference effect in multi-thread molecular junctions in between CNT leads of various cuts. To this purpose, we first show our calculations concerning the surface Green’s functions of cross-cut and angled-cut $(n,n)$ CNTs, as well as cross-cut $(n,0)$ CNTs, using both the iterative method and the integration method within the TB model. The results from both methods, in comparison with the bulk values, show the effect brought by the formation of different cuts. The contributions from bulk states and edge states can be differentiated by the comparison between the 2 methods. While the cross-cut $(n,n)$ CNTs present no edge states but oscillations of a 3-layer-cycle, the angled-cut cases exhibit both oscillations and the edge states in the vicinity of ${E}_{{\rm{F}}}$. Cross-cut $(n,0)$ CNTs present edge states, but no oscillations.

In the follow-up calculations for transmissions through molecular junctions in between angled-cut $\mathrm{(8,8)}$ CNT electrodes, we compare the *ab initio* results with the iterative method results. In the two-thread cases, the agreement between both approaches displays the effect of interference between the even and the odd channels. The discrepancy between the two approaches mainly results from overlooking the difference between the intra-molecular hopping and intra-CNT hopping, and is explained by our observation on the molecular bond lengths.

We also present a transmission study on one- and two-C_8_H_10_ junction in between cross-cut $(12,0)$ and $(13,0)$ CNT electrodes, where we focus on the comparison between the iterative method and the integration method. For the $n=3m$ cases, where the original bulk is gapless, the presence of edge states even takes away the transmission in the vicinity of the ${E}_{{\rm{F}}}$. Looking into the two-polyene cases with all possible combinations of contact sites on the (12, 0) CNT leads, we show that the interference effect is present via either path, and the comparison between both paths’ results reveals the edge state influence in the vicinity of E_F_.

As the fabrication of CNT-junction-CNT systems is becoming more developed and controlled, theoretical understanding of electronic structures at CNT edges, contact and molecule properties, and the interplay between the previous two aspects, are practically needed. Our study reveals the importance of the interference effect, through the discussion of the LDOS of CNT edges, and the interplay between the molecule and the leads via the contact selections.
